# 1.Q. Workshop: Summarising research evidence related to COVID-19 impact

**DOI:** 10.1093/eurpub/ckac129.059

**Published:** 2022-10-25

**Authors:** Organised by: PHIRI, Chair persons: Maria João Forjaz (Spain), Paulo Nogueira (Portugal)

## Abstract

After the first SARS-CoV-2 infections surge in late 2019, and its spreading worldwide, COVID-19 rapidly became a pandemic by March 2020. Facing a new public health crisis, the scientific community deployed research efforts to study this new disease, generating a large amount of scientific evidence in a very fast way. This research was developed in several directions, for instance, describing how COVID-19 impacts the population's health and well-being. Specifically, the research aimed at describing and evaluating the risk factors either for being infected or evolving toward adverse outcomes, such as hospitalisation or death. Moreover, the effectiveness of implemented public health measures was assessed since countries’ governments rapidly implemented them to mitigate the pandemic.Therefore, several literature reviews have been performed within the European Project ‘Population Health Information Research Infrastructure (PHIRI)’ to summarise and share the published COVID-19 evidence through a health information portal, where researchers and other stakeholders can exchange best practices and expertise. This workshop aims at sharing the collaborative work and experience of a large group of European researchers within the PHIRI consortium, consisting in preliminary results and lessons learnt with the scientific community. The workshop format consists of five presentations by PHIRI members, each one presenting their literature review work, followed by a discussion among the presenters and with the audience. The first workshop presentation will describe the methodological aspects of research conducted to assess the COVID-19 impact on the population's health and well-being, including research methods and statistical methods. The subject for the second talk will show a representative sample of health indicators used to evaluate the direct impact of COVID-19 in the scientific literature. The third presentation will elaborate on a short and long-term impact of COVID-19 crisis on population with frailty, multimorbidity or with different socioeconomic status; evidence derived from systematic literature reviews of population-based studies. The fourth presentation will describe the effectiveness and impact of tracking COVID-19 patients using digital contact tracing tools. The last presentation describes the methodological aspects of a systematic literature review conducted to gain an overview of the foresight studies that have been done throughout the World about COVID-19.

**Key messages:**

• During the pandemic, a literature reviews provide an important tool to summarise the large amount of scientific evidence that was generated.

• This workshop aims at sharing the collaborative work and experience of a large group of European researchers participating at PHIRI, the results and lessons learned with the scientific community.

